# Bipolar disorder and Quality of life assessment using the SF-12 health survey

**DOI:** 10.1192/j.eurpsy.2024.907

**Published:** 2024-08-27

**Authors:** N. Gannoun, G. Bahri, N. Mechergui, H. Ben Said, D. Brahim, M. Mersni, M. Bani, Y. Imene, N. Ladhari, M. Methni, C. Ben Said, N. Bram

**Affiliations:** ^1^Occupational Medicine health department, Charles Nicolle hospital; ^2^Forensic psychiatry départment, Razi hospital, la Manouba, Tunis, Tunisia

## Abstract

**Introduction:**

Bipolar disorder (BD) is a severe and chronic mental illness characterized by recurrent major depressive episodes and mania (BD-I) or hypomania (BD-II). In addition to the burden of the disease and its consequences, people living with BD, like many other people suffering from mental illness, must deal with their difficulty of integration which can influence their personal and professional life and consequently their quality of life (QOL).

**Objectives:**

The aim of our study is to assess the QOL among working patients with BD.

**Methods:**

A cross-sectional study was carried out in the occupational medicine department of the Charles-Nicolle hospital in Tunisia. Sociodemographic and occupational data were collected from the medical records of patients with bipolar disorder who consulted our department during the period 2022 to 2023. and a telephonic survey was carried out to complete the SF 12 international scale, which is a general health questionnaire that consists of 12 questions which investigates the patient’s state of health via 8 different dimensions: General health perception, Physical health, Limited physical role function, Physical pain, Vitality, Mental health, Limited emotional role function and social functioning.

**Results:**

We enrolled a total of 46 cases where 76% with BD type 1 with an average age of 43±9 years. Most participants were female (76%) and the most frequent sectors of activity were healthcare and administration (80% and 12% respectively). BD was well balanced in 39% of cases with an average bipolar history of 7 years. The median annual absence due to psychiatric problems was 92±61 days per year. The average score was 44±18 for the General Health, 57±35 for physical health and 67±18 for mental health.

**Conclusions:**

This study revealed that people living with BD’s QOL seems to be altered. Clinicians need to be attentive to the QOL of their patients, its assessment, and its empowerment in their daily clinical practice. Future work is required to establish valid strategies to fight low QOL among patients suffering from BD.

**Disclosure of Interest:**

None Declared

